# Feline Infectious Peritonitis as a Systemic Inflammatory Disease: Contribution of Liver and Heart to the Pathogenesis

**DOI:** 10.3390/v11121144

**Published:** 2019-12-10

**Authors:** Alexandra J Malbon, Sonja Fonfara, Marina L Meli, Shelley Hahn, Herman Egberink, Anja Kipar

**Affiliations:** 1 Institute of Veterinary Pathology, Vetsuisse Faculty, University of Zurich, 8057 Zurich, Switzerland; anja.kipar@uzh.ch; 2 Center for Clinical Studies, Vetsuisse Faculty, University of Zurich, 8057 Zurich, Switzerland; mmeli@vetclinics.uzh.ch; 3 Department of Clinical Studies, Ontario Veterinary College, University of Guelph, Guelph, ON N1G 2W1, Canada; sfonfara@uoguelph.ca; 4 Small Animal Hospital, Faculty of Veterinary Medicine, University of Helsinki, 00014 Helsinki, Finland; 5 Department of Basic Veterinary Sciences, Faculty of Veterinary Medicine, University of Helsinki, 00014 Helsinki, Finland; shelley.hahn@tufts.edu; 6 Clinical Laboratory, Vetsuisse Faculty, University of Zurich, 8057 Zurich, Switzerland; 7 Virology Division, Department of Infectious Diseases and Immunology, Faculty of Veterinary Medicine, Utrecht University, 3584 CL Utrecht, The Netherlands; h.f.egberink@uu.nl

**Keywords:** feline infectious peritonitis, feline coronavirus, hepatocytes, cardiomyocytes, inflammatory cytokines, pathogenesis, systemic inflammatory response

## Abstract

Feline infectious peritonitis (FIP) is a fatal immune-mediated disease of cats, induced by feline coronavirus (FCoV). A combination of as yet poorly understood host and viral factors combine to cause a minority of FCoV-infected cats to develop FIP. Clinicopathological features include fever, vasculitis, and serositis, with or without effusions; all of which indicate a pro-inflammatory state with cytokine release. As a result, primary immune organs, as well as circulating leukocytes, have thus far been of most interest in previous studies to determine the likely sources of these cytokines. Results have suggested that these tissues alone may not be sufficient to induce the observed inflammation. The current study therefore focussed on the liver and heart, organs with a demonstrated ability to produce cytokines and therefore with huge potential to exacerbate inflammatory processes. The IL-12:IL-10 ratio, a marker of the immune system’s inflammatory balance, was skewed towards the pro-inflammatory IL-12 in the liver of cats with FIP. Both organs were found to upregulate mRNA expression of the inflammatory triad of cytokines IL-1β, IL-6, and TNF-α in FIP. This amplifying step may be one of the missing links in the pathogenesis of this enigmatic disease.

## 1. Introduction

Feline infectious peritonitis (FIP) is a coronavirus-induced fatal immune-mediated disease in cats, characterised by serofibrinous and granulomatous serositis, often with protein-rich effusions into body cavities, granulomatous phlebitis and periphlebitis, and granulomatous inflammatory lesions in several organs [1,2,3,4]. The disease presents clinically with recurrent fever and signs reflecting the distribution of organ lesions. The development of FIP lesions is triggered by activated, virus infected monocytes. In the presence of a generalised activation of venous endothelial cells, these monocytes induce the granulomatous phlebitis that is considered to be the first and hallmark lesion and can occur in a range of organs [4]. Endothelial cell activation, together with other systemic changes such as fever, indicates excessive systemic cytokine release though the precise sources remain unclear [4,5,6].

Pro-inflammatory cytokines are the main mediators of the innate immune response, allowing communication between and priming of the various components of the innate immune system, e.g., activation of leukocytes and endothelial cells (reviewed by [6,7]). In cats, interleukin (IL)-1β, IL-6, and tumour necrosis factor (TNF)-α are the main mediators of the acute phase inflammatory response [8] and cats with FIP exhibit clear clinical and histological evidence of overt inflammatory processes. Despite this, there are conflicting results on the presence and systemic levels of these cytokines in FIP. Early studies found high IL-1 and IL-6 activity in sera, ascitic fluid, and the supernatant of cultured peritoneal exudate cells of cats with FIP [9,10]. However, IL-6 mRNA levels in peripheral blood mononuclear cells (PBMC) were found to be unaltered, only mildly increased, or variable in FIP [11,12,13], whilst IL-10 and IL-12 transcription was markedly depressed [11].

In vitro transcriptome studies have also not shown these classical inflammatory mediators to be significantly altered between feline infectious peritonitis virus (FIPV) infected and uninfected cells; however, these have focussed on cell culture experiments using CRFK cells, so may not reflect the complex immune interactions in vivo [14,15]. Additionally, CRFK cells are not primary immune cells so are unlikely to exhibit the same response. In vitro studies using peritoneal macrophages, a system that is closer to the disease scenario, provided evidence of the importance of TNF-α. FIPV induced TNF-α production in infected cells; as TNF-α can itself upregulate the type II FCoV receptor aminopeptidase N, cells may thus enhance their own infection rate [16,17]. In turn TNF-α functions as a possible contributor to the lymphocyte apoptosis observed in FIP [18,19]. Despite this, in natural disease, end stage FIP is associated with only a low increase or in some cases even a decrease in TNF-α transcription in the lymphatic tissues themselves [20,21]. In conjunction, IL-1β and IL-6 transcription showed no to low increases in the lymphatic tissues in FIP whilst IL-10 and IL-12 decreased or remained unchanged [20,21].

These previous results suggest that lymphatic tissues are not by themselves mass producing inflammatory cytokines at the levels required in order to contribute to and potentiate a systemic activation of endothelial cells and monocytes, and that other organ systems may play a role. We therefore first hypothesised a role for hepatocytes in cytokine production, as these have already been shown to be active in the disease. In cats with FIP, hepatocyte produced acute phase proteins such as haptoglobin, serum amyloid A, and in particular alpha1-acid glycoprotein (AGP) have been shown to be elevated; to the extent that they are considered key elements of the diagnostic process [22,23,24]. Although not thus far studied in felines, human and murine hepatocytes have also been shown capable of producing cytokines, including IL-1, IL-6, IL-10, IL-12, and TNF-α [25,26,27,28,29,30]. Additionally, our group has previously identified constitutive pro-inflammatory cytokine expression by feline cardiomyocytes, which increases in systemic inflammatory disease [31]. This led to the second hypothesis that the heart is an additional source of cytokines and thereby contributes to the systemic inflammatory status that allows FIP lesions to develop.

To evaluate these hypotheses, we measured relative mRNA levels of IL-1β, IL-6, and TNF-α in liver and heart by quantitative reverse transcriptase polymerase chain reaction (RT-qPCR). We then used a palette of methods on selected cases to confirm translation and identify the cell source of the cytokines. In light of both our previous findings in lymphatic tissues [20,21] and the importance of these cytokines in determining the balance of the immune response (Th1 vs. Th2/pro- vs. anti-inflammatory [32,33]), we also evaluated the hepatic IL-10 and IL-12p40 mRNA levels in an attempt to assess whether this ratio correlated with the clinical picture.

We demonstrated that both hepatocytes and cardiomyocytes are sources of inflammatory cytokines in FIP, and that the hepatic IL-12:IL-10 balance is skewed towards IL-12 in diseased animals.

## 2. Materials and Methods

### 2.1. Animals and Tissue Processing

#### 2.1.1. Liver Study (Groups 1.1–1.3)

This study was undertaken on three groups of cats. Group 1.1 comprised 16 cats with FIP, further subdivided into natural infection (12 pet cats; age: 5 months to 2 years; Group 1.1a) and experimental infection (four female specific pathogen free (SPF) cats; age: 14–16 weeks; Group 1.1b), see Table 1. All 1.1a cats were submitted for diagnostic post mortem examination with full owner consent. The 1.1b cats had been euthanased with FIP after experimental intra-peritoneal infection with the serotype I FCoV strain FIPV-UCD at the University of Utrecht, The Netherlands. Approval for this experiment was obtained from the Ethical Committee of Utrecht University (approval number: 0502.0802). All Group 1.1b cats showed clinical signs of FIP which necessitated euthanasia of two cats at 3.5 and 4 weeks post infection (p.i.) whilst the remaining two were euthanased at the end of the experiment (11 weeks p.i.). 

The diagnosis of FIP was confirmed in all cases by gross, histological, and immunohistological examination [3]. Six of the 16 cats with FIP had effusions, including three of the four experimental cases (data was unavailable for three animals).

Group 1.2 consisted of 14 clinically healthy, male SPF cats that had been per-orally infected at an age of 8.5 to 27 weeks with previously isolated serotype I FCoV field strains of enteric pathotype (FCoVZu1, 2, 3, and 5 -feline enteric coronavirus; FECV) and had been euthanased between 2 and 12 weeks p.i. [34]. This experiment was performed under the Swiss regional legislation (project license number TVB 66/2000). All cats had tested positive for FCoV shedding and those euthanased more than 2 weeks p.i. seroconverted. All were confirmed to be systemically FCoV infected by the presence of a FCoV viraemia [34]. These cats were used as a comparison group to provide relatively uniform baseline cytokine level as pet cats without FIP would be subject to wide variations in terms of pathogen exposure, FCoV infection status, and concurrent disease. This also allowed evaluation of the effect of FIP on the animal rather than FCoV infection per se.

All group 1.1 and 1.2 animals were necropsied within 1 h of death. Liver samples from grossly normal regions (i.e., without FIP lesions) were collected and immediately frozen at −80 °C for RNA extraction, whilst normal and lesion samples were fixed in 10% buffered formalin for 24–48 h and routinely paraffin wax embedded for histological and immunohistological examination. 

The third group (Group 1.3) comprised six healthy untreated SPF cats, aged 36–38 months, that had been euthanased at the University of Glasgow, UK as part of a study performed under UK Home Office Project Licence PPL 60/3735. From these cats, formalin fixed, paraffin embedded liver samples were kindly provided by Prof M Hosie. 

#### 2.1.2. Heart Study (Groups 2.1–2.3)

This study was undertaken on three additional groups of cats. Group 2.1 comprised 18 pet cats (age: 2 months to 3 years; mean age: 14 months) that had died or were euthanased with FIP; the diagnosis was confirmed as described above. See Table 2.

Group 2.2 comprised 10 cats that had been euthanased due to non-inflammatory diseases not expected to have any systemic impact (24 months to 19 years; mean age: 9 years) which were further grouped by age (Group 2.2a (*n* = 4), (two each); Group 2.2b (*n* = 6), aged 9–19 years, mean age: 13.4 years) to acknowledge the fact that age has an effect on constitutive cytokine expression in the myocardium [35]. See Table 2.

Group 2.3 comprised three cats with systemic inflammatory diseases other than FIP. See Table 2.

All cats had been euthanased and submitted for diagnostic post mortem examination with full owner consent. They were necropsied within 1 h of death. Pleuritis involving the outer pericardium was observed in one of the FIP cats, however, neither this cat nor any of the others exhibited any gross changes in the heart. 14 of the 18 cats with FIP had effusions (data was unavailable for one animal). Hearts were removed and samples collected from both atria, both ventricular free walls, and the interventricular septum into RNA*later*^TM^ Stabilization Solution (Thermo Fisher Scientific, Ilkirch Cedex, France) and stored at −80 °C until RNA extraction. The remaining heart was fixed in 10% buffered formalin for 24–48 h, trimmed, routinely paraffin wax embedded and subjected to a histological examination. This did not reveal pathological changes in the myocardium of any of the cats.

### 2.2. Assessment of Cytokine Transcription

#### 2.2.1. Reverse Transcription and Quantitative Polymerase Chain Reaction (RT-qPCR) for Feline Cytokines

From each frozen liver and heart sample, approximately 50–100 mg was taken for RNA extraction and subsequent cDNA synthesis, following published protocols [21,34]. For the liver, RT-qPCR for feline IL-1β, IL-6, IL-10, IL-12p40, and TNF-α was carried out on the cDNA samples, using previously published assays [21,36,37]. For the heart, IL-1β, IL-6, and TNF-α transcription was assessed. The mRNA levels of the housekeeping gene feline glyceraldehyde-3-phosphate dehydrogenase (GAPDH) served as internal controls. All reactions were run in duplicate, using previously published amplification conditions and assay compositions [21,35,37]. Briefly, the thermal profile was as follows: 50 °C for 2 min, 95 °C for 10 min, and 45 cycles of 95 °C for 10 s, and 60 °C for 1 min. Data collection occurred during the extension phase (60 °C). 

#### 2.2.2. Laser Capture Microdissection (LCM)

This was performed as proof of principle on a single FIP cat heart (Group 2.1) to confirm that cardiomyocytes are themselves responsible for the detected mRNA transcription, and was therefore not subject to any statistical analysis. 

Frozen RNA*later*^TM^ samples were embedded in Tissue-Tek^®^ O.C.T. Compound (Sakura^®^ Finetek USA Inc., Alphen aan den Rijn, The Netherlands) in a cryostat at −20 °C before sectioning at 8–10 µm onto PEN Membrane Glass Slides (Applied Biosystems™, Waltham, MA, USA) and staining according to published protocols ([38]). The slides were completely air dried before LCM to allow for proper excision performance. 

The ArcturusXT™ Laser Capture Microdissection System (Thermo Fisher Scientific) and Arcturus^®^ CapSure^®^ Macro LCM Caps (Thermo Fisher Scientific) were used for the LCM process itself. Areas of myocardium with highly enriched populations of cardiomyocytes (i.e., avoiding all small to large vessels and adipocytes) were identified and isolated. The isolation of cells of interest was verified by microscopic examination of the LCM cap as well as of the excised region after microdissection.

The Qiagen RNeasy Microkit (Qiagen, Hilden, Germany) was used for RNA extraction as per the manufacturer’s protocol; briefly, the LCM cap was placed on a 500 µL Eppendorf tube containing extraction buffer and incubated inverted at 42 °C for 30 min before proceeding to the next steps. From the final elute of 12 µL of RNA, 8 µL was used for cDNA synthesis using the SuperScript IV VILO Master Mix kit (Thermo Fisher Scientific) with an integrated DNase step to remove traces of genomic DNA. This was followed by target specific pre-amplification using the primers described above and the TaqMan^®^ PreAmp Master Mix (2 ×) (Applied Biosystems™) to produce sufficient cDNA for qPCR analysis. The pre-amplification was performed according to the manufacturer’s protocol using 20 PCR cycles. The resulting product was then diluted 1:20 and used for all RT-qPCR reactions as described above. 

#### 2.2.3. Relative Quantification of Cytokine Transcripts and Statistical Analysis

Relative quantification of cytokine signals was done by the comparative threshold cycle (C_T_) method [39] using fGAPDH as the internal reference gene. This serves to normalise for differences in the amount of total nucleic acid added to each reaction and the efficiency of the reverse transcriptase step [21,37]. The programme IBM SPSS^®^ was used for statistical comparisons; a two-tailed Mann–Whitney test was applied at the 95% confidence level on the premise that cytokine levels in the two groups were different.

In the liver study (1), first the natural FIP cases (Group 1.1a) were compared with the experimental FIP cases (Group 1.1b) to determine if they could be classed as one group. As no statistically significant difference was found between these subgroups, all of Group 1.1 was compared to Group 1.2. Both Group 1.1a and 1.1b were also compared separately to Group 1.2. Within Group 1.1, cytokine mRNA levels from cats with and without effusions were compared. Each disease form subgroup of FIP cats was then compared with control cats. Where clinical information regarding the presence of effusions was lacking, the individuals were excluded from these comparisons. The IL-12p40:IL-10 mRNA ratio was evaluated using the Independent Samples Median Test.

In the heart study (2), cats with FIP (Group 2.1) were compared to control cats (Group 2.2) and cats with systemic inflammatory disease (Group 2.3). To take a potential age influence into account, FIP cats and cats with systemic diseases were subsequently each compared to young (Group 2.2a) and old (Group 2.2b) control cats. Within Group 2.1, FIP cases were split into those with and without effusions. These were first compared to each other and then separately to control cats, age subgroups of the control cats, and cats with systemic inflammatory diseases.

### 2.3. Assessment of Cytokine Protein Expression

#### 2.3.1. Antibodies Specific for Feline Cytokines

Since antibodies reacting with the feline cytokines of our interest were not available, a panel of rabbit anti-peptide antibodies, specific for feline IL-1β, IL-6, IL-10, IL-12p40, and TNF-α was commercially produced (Genosphere Biotechnologies, Paris, France). Antibodies were raised against the C-terminus of the proteins by using 14 amino acid long synthetic peptides with an additional cysteine residue at the N-terminus coupled to keyhole limpet hemocyanin (KLH) carrier (see supplemental Table S1). All antisera had been determined by the supplier by ELISA to have a titre greater than 1:10,000 when tested against the peptide antigen. Antibody specificity was confirmed by ELISA and dot blot, using recombinant cytokines where available (human IL-1β and TNF-α (Peprotech, London, UK); feline IL-6 (Serotec (now Bio-Rad, Oxford, UK)); feline IL-1β, IL-10, TNF-α, IL-12 (R&D Systems, Zug, Switzerland), and following routine protocols. Immunoblotting was also performed following routine protocols on a homogenised liver sample from an experimental FIP cat to confirm reaction with the ‘native’ protein. The antibodies were subsequently used in immunohistology. As IL-6 specificity could not be confirmed in the Western blot system, a mouse monoclonal antibody directed against feline IL-6 (MAB23051, R&D Systems) that became commercially available following completion of the initial liver study was tested on a small set of liver samples. This antibody has been previously published [40] and served here to further confirm IL-6 protein expression in the myocardium.

#### 2.3.2. Immunohistology for Feline Cytokines

Staining protocols for the anti-peptide antibodies were optimised using FIP lesions from a diagnostic case, in which a mixed inflammatory cell population was present. The staining was then applied to a selection of cases (five cats from Group 1.1a, all four cats from Group 1.1b, all cats from Group 1.3) from the liver study (1). Briefly, formalin-fixed, paraffin embedded liver sections (3–5 µm) were subjected to immunohistological staining following the previously published peroxidase anti-peroxidase (PAP) method [3], with diaminobenzidine as the chromogen and Papanicolaou’s haematoxylin counterstaining. Sections incubated with rabbit pre-immune serum and/or PBS instead of the primary antibody, and sections incubated with antibodies blocked by the respective peptide served as negative controls. These did not yield any reaction. Stained liver sections were assessed independently by two pathologists (AJM and AK) and staining was graded in a semi-quantitative manner as negative, weak, moderate or strong, based on the number of positive hepatocytes and average staining intensity.

For the heart study (2), formalin-fixed, paraffin embedded sections from the left and right ventricular free wall and atrium and from the interventricular septum were stained for IL-6, using the commercial IL-6 antibody (see above; the custom-made antibodies were no longer available at this time). Briefly, protocols were as follows: pre-treatment with citrate buffer followed by 15 min serum block, incubation with primary antibody (diluted 1:500 in dilution buffer (Dako, Basel, Switzerland; S2022) at room temperature for 1 h, use of the EnVision Mouse (Dako Cytomation) detection system. Chromogen, counterstaining methods, and controls were as described above.

## 3. Results

### 3.1. Inflammatory Cytokine Transcription in the Liver

Hepatic transcription of all tested cytokines was detectable in all groups, and in the majority of animals (81–100%, cytokine dependent; Table S2).

**Figure 1:**
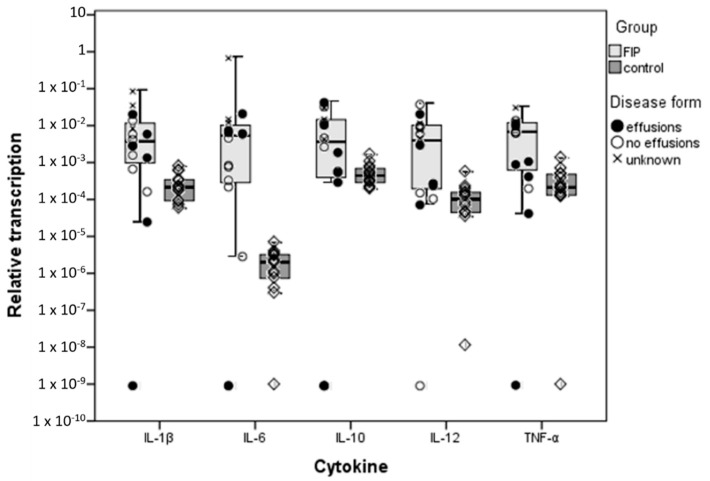
Comparison of relative cytokine transcription levels in the liver between cats with feline infectious peritonitis (FIP) and healthy, feline coronavirus (FCoV)-infected cats; box and whisker plots together with illustration of individual cat values and presence or absence of effusions in the case of FIP. ‘FIP’ includes Group 1.1a–natural FIP cases in the left-hand column of the box, and 1.1b–experimentally infected cats with FIP at the right-hand side; ‘control’ cats are Group 1.2–FCoV-infected cats without FIP. Boxes indicate the median value and the interquartile range, whilst whiskers indicate the spread of values with the exception of outliers (calculated by SPSS as >1.5 box lengths).

Relative transcription levels of all cytokines were significantly higher (*p* < 0.05) in cats with FIP. Variation between animals was observed in all groups, this being greater in the FIP group (Figure 1). In the latter, the within group variation for each cytokine was lower in the experimentally infected group (1.1b) than in natural infection (Group 1.1a), and so were the transcription levels overall (though not significantly so) (Table S2). When comparing naturally and experimentally infected cats with FIP separately with the FECV-infected healthy cats, a significant difference was observed for the experimentally infected animals with FIP only for IL-6 which was transcribed at a higher level in cats with FIP (Figure 1; Table 3).

The IL-12p40:IL-10 mRNA ratio was assessed as an accepted indicator of the inflammatory balance of the immune system [32,33]. This was overall significantly higher in cats with FIP (*p* = 0.047), indicating the balance is tipped towards a pro-inflammatory state. In fact, IL-10 levels were higher than IL-12 levels in each individual control cat, whereas in half of the naturally infected FIP cats IL-10 levels were lower than IL-12 levels. Experimental FIP cats were again in between the groups, with slightly higher IL-10 than IL-12 levels in all animals.

Il-6 showed the largest quantitative difference in medians between cats with and without FIP, with mRNA levels nearly 1000 fold higher in disease. Interestingly, this was owing to lower relative transcription levels for IL-6 in healthy cats than of the other cytokines (though the difference was not statistically significant), whereas in cats with FIP, IL-6 levels were on a par with those of other cytokines. IL-6 was also the only cytokine that varied depending on the presence or absence of effusions; its transcription was significantly higher (*p* = 0.04) in cats with effusions than in those without (Table 3). For IL-12 and TNF-α, a close to 100 fold increase in relative transcription was seen between cats with and without disease, whilst the smallest quantitative difference was found for IL-1β and IL-10; for both cytokines, mRNA levels were only ~10 fold higher in FIP.

### 3.2. Hepatocytes Are a Source of Inflammatory Cytokines in FIP

**Figure 2:**
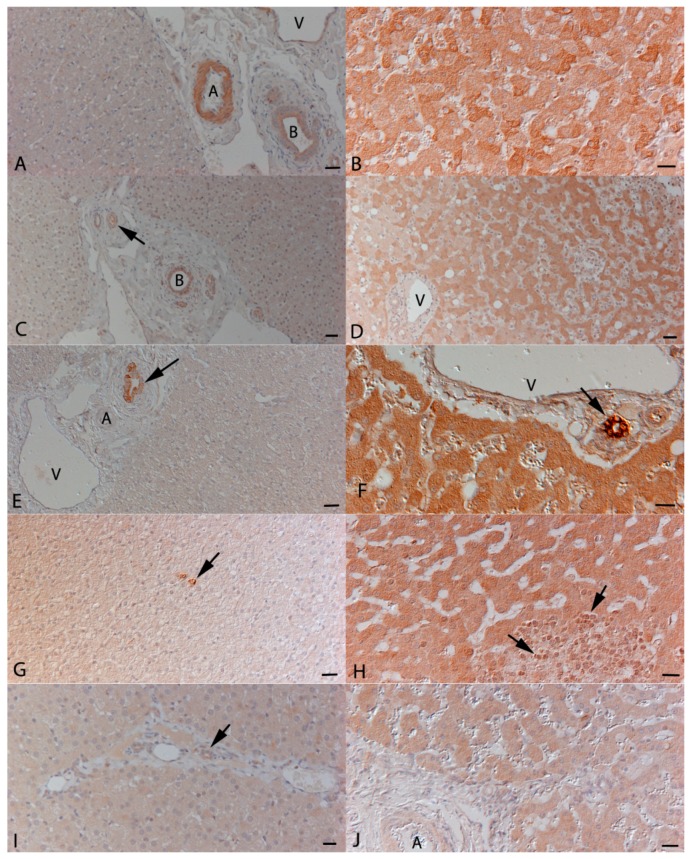
Representative immunohistological staining for the expression of cytokines in the liver. Left column: specific pathogen free (SPF) cats; right column: cats with FIP. (**A**,**B**): Expression of IL-1β. (**A**): In SPF cats, expression is restricted to bile duct (B) epithelial cells and the media of small arteries (A). V: vein in portal area. (**B**): In a FIP cat, hepatocytes exhibit variably intense expression. (**C**,**D**): Expression of IL-6. (**C**): In SPF cats, expression is restricted to a weak staining in bile duct (B) epithelial cells and the media of small arteries (arrow). (**D**): In a FIP cat, a large proportion of hepatocytes exhibit variably intense expression. V: vein in portal area. (**E**,**F**): Expression of IL-10. (**E**): In SPF cats, expression is restricted to bile duct epithelial cells (arrow). A: artery, V: vein in portal area. (**F**): In a FIP cat, hepatocytes exhibit a diffuse strong expression. The staining in bile duct epithelial cells (arrow) is even stronger. V: vein in portal area. (**G**,**H**): Expression of IL-12p40. (**G**): In a SPF cat, weak expression is seen within hepatocytes. Occasional positive Kupffer cells (arrow) are also seen. (**H**): In a FIP cat, hepatocytes exhibit a diffuse weak to moderate expression. In a granulomatous infiltrate, there are some weakly positive macrophages (arrows). (**I**,**J**): Expression of TNF-α. (**I**): In a SPF cat, very weak expression is seen within hepatocytes and bile duct epithelial cells (arrow). (**J**): In a cat with FIP, hepatocytes exhibit a diffuse weak staining. Bars represent 50 µm (A, C, D, E, and G) or 20 µm (B, D, H, I, and J) Peroxidase anti-peroxidase method, Papanicolaou’s haematoxylin counterstain.

Immunohistology was then used to identify the cell sources of the cytokines. The SPF cat livers were histologically unaltered and served to assess FCoV-independent constitutive protein expression. Cytokine expression was mainly evident in the bile duct epithelium which exhibited variable expression of all cytokines (Figure 2). There was also occasional evidence (i.e., in one or two animals for each cytokine) of very low level expression by hepatocytes, represented by a weak, finely granular cytoplasmic staining (Figure 2G,I). Furthermore, Kupffer cells in all SPF cats occasionally expressed IL-1β and in two and one cat respectively also IL-6 and IL-12 (Figure 2G).

The livers of FCoV-infected cats without FIP often exhibited mild portal lymphocyte infiltration as well as occasional small clusters of lymphocytes and scattered individual neutrophils within the sinusoids. The cytokine expression pattern and intensity were similar to that seen in SPF cats; hepatocytes largely exhibited either no or very weak staining.

In cats with FIP, the majority of livers exhibited typical lesions, i.e., fibrinosuppurative perihepatitis and/or focal to multifocal granulomatous-necrotising inflammation. Cytokine expression by inflammatory cells was generally weak, but all cytokines were found to be expressed by macrophages and neutrophils as well as low numbers of Kupffer cells (Figure 2H). Hepatocytes were shown to express all cytokines. This was seen either as a diffuse staining of all hepatocytes (Figure 2F,H,J) or appeared to vary in its intensity between individual cells (Figure 2B,D). A direct correlation between protein expression and mRNA levels was not observed by semi-quantitative assessment of cytokine expression.

### 3.3. Inflammatory Cytokine Transcription in the Heart

IL-1β, IL-6, and TNF-α were all constitutively transcribed in the heart.

**Figure 3:**
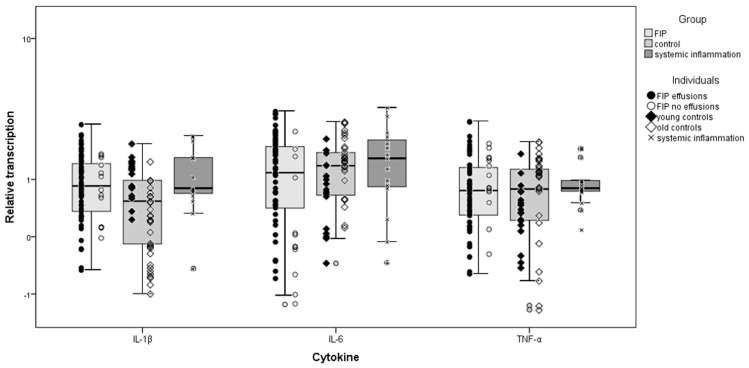
Comparison of relative cytokine transcription levels in the heart between cats with feline infectious peritonitis (FIP), control cats, and cats with systemic inflammatory disease other than FIP; box and whisker plots together with illustration of individual cat values. Group 2.1, FIP cases; 2.2, control cats (young ≤3 years old; old ≥9 years old); 2.3, systemic inflammatory disease. Boxes indicate the median value and the interquartile range, whilst whiskers indicate the spread of values with the exception of outliers (calculated by SPSS as >1.5 box lengths).

Cats with FIP exhibited significantly higher myocardial IL-1β (*p* = 0.008), IL-6, and TNF-α (both *p* < 0.001) transcription than control cats. When control cats were split by age, older cats retained the significant differences for all three cytokines (*p* < 0.001) whereas only TNF-α was significantly lower in the young control cats than in cats with FIP (Table 4). There were no significant differences between cats with systemic inflammatory diseases and FIP or between cats with FIP with and without effusions., These FIP subgroups (with and without effusions) were compared in turn to the other groups, with results almost identical to those obtained using the combined FIP group. The exception was in comparing FIP cats without effusions to controls, which were not significantly different despite the combined FIP group and FIP cats with effusions showing significantly higher transcription levels than controls (displayed graphically in Figure 3). Data distribution is summarised in Table S3.

### 3.4. Cardiomyocytes Themselves Contribute to Cytokine Expression

Laser capture microdissection samples of myocardium demonstrated mRNA expression of IL-1β, IL-6, and TNF-α in cardiomyocytes.

Staining of a representative selection of heart samples from cats with FIP for IL-6 confirmed IL-6 production predominantly by cardiomyocytes. Endothelial cells and smooth muscle cells of the tunica media were also positive in some sections and mesothelial cells showed irregular staining.

## 4. Discussion

Here we present the first functional studies of their kind to focus on the potential contribution of non-immune organs, i.e., the liver and heart, to cytokine production in cats affected by FIP and, more generally, by systemic inflammatory disease per se. The specific panel of cytokines, IL-1, IL-6 and TNF-α as well as IL-10 and IL-12p40, was chosen based on their known effects and to assess their potential role in disease manifestation, as FIP is driven by activated monocytes and characterised by vascular inflammatory lesions and fever.

Using a combination of methods to demonstrate cytokine transcription and in situ protein expression as well, we could show that both organs produce the entire panel of pro-inflammatory and immunomodulatory cytokines, and confirmed the parenchymal cells, hepatocytes and cardiomyocytes, as a source of these.

The varying origins of the different groups of cats reflects the availability of tissue samples. The study aimed to maximise the information gleaned from previous unrelated experimental infections and case collections by utilising these tissues to tackle the hypotheses. Results obtained from the liver study provided a springboard to studying the heart, for which retrospective samples were available. They are presented together here as it was felt that, combined, the results add more to our knowledge of potential pathogenesis. This of course comes with the limitation of inter-group matching, hence we have drawn broad rather than highly specific conclusions and the groups have not been compared between organs.

In the liver, there was a low level of expression observed in healthy FCoV-infected cats, whilst the transcription of IL-1β, IL-6, IL-10, IL-12, and TNF-α was significantly upregulated in FIP. Interestingly, when comparing natural and experimental FIP, the range of cytokine transcription extended higher in the natural cases. It is possible that the prior SPF status of the experimentally infected cats accounts for this as they would not have had previous exposure to FCoVs and/or other common infectious pathogens. They would also be at a more regulated stage of disease and received identical virus and dosage, likely explaining the lower variability of the results. This difference between groups, with higher levels in many of the natural cases, also supports the role of these organs in systemic inflammation generally rather than as a FCoV specific response.

In cats with FIP, not only hepatocytes but also inflammatory cells within the typical macrophage dominated lesions were found, unsurprisingly, to express the examined cytokines. However, the inflammatory cells likely played a minor role in the overall hepatic transcription, since we extracted RNA from tissue specimens without grossly visible FIP lesions and generally found immunohistological evidence of more consistent cytokine expression by hepatocytes than by inflammatory cells. The different antibodies showed variation in their staining patterns, being either relatively diffuse or showing more varying intensity between hepatocytes. The precise cause of this is unknown but may reflect variations in cytokine expression and release beyond the scope of this study.

The heart was not affected by FIP lesions in any case, which is consistent with the known lesion distribution [41]. Despite this, the pyrogenic cytokines IL-6 and TNF-α were upregulated in FIP; this was also seen in other systemic inflammatory diseases, these likely affected the heart in a similar way. Cardiomyocytes were confirmed to be a source of both cytokine mRNA and protein.

These findings suggest that ‘bystander’ cells such as cardiomyocytes and hepatocytes are likely to play a significant role in amplifying systemic inflammation by responding non-specifically to inflammatory processes. Immunohistological staining results did not allow an accurate quantification of translation, nor could we identify a direct correlation between mRNA and protein levels. This has a number of possible explanations—the different regions sampled, the different sensitivities of the two methods, the time lapse between transcription and translation (with our samples all taken at a single time point), and the manifold levels of pre- and post-translational regulation to which cytokines are exposed. Taking TNF-α alone, there is regulation of mRNA at export, post-transcription, and translation, with multiple mechanisms at each stage [42]. However, our objective was to confirm rather than quantify parenchymal cytokine expression and, together with what is known of cytokine function, our results provide strong evidence that hepatocytes and cardiomyocytes may indeed play a role in systemic inflammatory disease and hence the pathogenesis of FIP. This is supported by data from a mouse study showing that hepatocytes can produce at least as much IL-6 per cell as macrophages [28]. Traditionally thought to have more of a reactive or even bystander role in disease, the parenchymal cells may in fact provide a large amplifying step in a cascade of cytokine release. This role is, however, secondary and rather unspecific, as systemic inflammatory diseases were found to be associated with myocardial transcription of IL-1β, IL-6, and TNF-α at levels similar to those seen in cats with FIP. Whether other organs such as the lung (in which resident macrophages such as pulmonary intravascular macrophages may be targets for FCoV [43]) also have a role to play remains to be investigated.

IL-1β, IL-6, and TNF-α are the major inflammatory cytokines in cats; in FIP, they can be linked to both lesion induction and clinical signs such as fever. IL-1β and TNF-α may also contribute to the hypoalbuminaemia and weight loss seen in FIP via decreased albumin production and increased muscle breakdown respectively [8,44]. Adhesion molecule expression and chemokine production by endothelial cells (EC) is induced by TNF-α and IL-1β, leading to leukocyte binding [45]. In line with this, upregulation of β2-integrins was seen on leukocytes from cats with FIP [4,46]. The cytokines can then be expected to contribute to the destruction of the vascular basal lamina seen in FIP phlebitis [1,3,4,47], as they upregulate matrix metalloproteinase secretion from monocytes [48,49].

IL-6, known to be particularly stimulated by IL-1β and TNF-α [50], showed the greatest hepatic upregulation of the studied cytokines in association with FIP and was the only one to be significantly higher in the effusive form of disease compared to the non-effusive form. In wet FIP, significant IL-6 activity has previously been reported in sera and peritoneal exudate cells [9]. The wet, dry, and mixed forms of FIP indicate overlapping disease states with a variable degree of vascular permeability [51,52]. IL-6 can induce vascular endothelial growth factor (VEGF) production and pulmonary vascular permeability [53,54,55], and the degree of ascites was found to be correlated with serum VEGF levels in cats with FIP [51]. This could explain, via VEGF upregulation, the particularly high hepatic IL-6 transcription levels seen in FIP cats with effusions in our study. Hepatic IL-6 release could also contribute to the progressive plasma cell infiltration seen in older FIP lesions [3,41], as IL-6 induces the final maturation of B cells into plasma cells [56]. It might also influence myocardial cytokine transcription, which showed a lower increase in FIP when compared to control cats than the liver samples and might therefore suggest a later, reactive involvement of the myocardium in the disease process. Showing a reactive response might also be the reason that no difference in myocardial transcription was observed between the effusive and non-effusive forms of FIP. However, further investigations into the time course of FIPV infection are needed.

Interestingly, IL-1β has been shown experimentally to more than double the half-life of IL-6 mRNA, and TNF-α had a similar, but less intense effect [57]. Accordingly, in addition to its direct effects, IL-1β could have two relevant synergistic effects in FIP; stimulating IL-6 transcription and prolonging the lifespan of IL-6 mRNA [50], thereby potentiating its effects. The similar IL-1β and IL-6 mRNA concentrations in the myocardium of young control cats and cats with FIP confirms our previous finding of a generally more reactive/inflammatory myocardium in young cats [35], which might also contribute to the increased susceptibility of young cats to FIP, with TNF-α subsequently further amplifying the inflammatory process.

IL-10 is an anti-inflammatory and immunosuppressive cytokine that inhibits inflammatory cytokine transcription by monocytes, but also complements IL-6 as it can stimulate antibody production [58,59]. The host’s attempt to counteract the pro-inflammatory signals in FIP through increased IL-10 expression may therefore have the negative side effect of promoting anti-FCoV antibody production.

We also found IL-12 upregulation in the liver in association with FIP. The pro-inflammatory effects of IL-12 are usually counteracted by IL-10 which attempts to return the immune response to homeostatic base levels [60]. The IL-12:IL-10 ratio has been used as a measure of the balance between pro- and anti-inflammatory states in a number of disease conditions [61]. In our study we found an overall higher hepatic IL-12:IL-10 mRNA ratio in FIP than in healthy FCoV-infected cats. Despite being archetypally pro-inflammatory, at high concentrations IL-12 can inhibit the immune response and the generation of cytotoxic T lymphocytes [62,63]. In contrast to the liver, the mesenteric lymph nodes were previously found to downregulate IL-12 transcription in FIP, with IL-10 levels unaffected [20,21]. It would appear that neither high IL-12 nor high IL-10 are themselves protective and indeed plasmids encoding IL-12 have been found to enhance susceptibility to disease in FIPV vaccination studies, rather than offering protection as had been predicted [64].

## 5. Conclusions

This study sheds light on the contribution of non-immune organs, specifically the heart and liver, to systemic disease in which they are not directly targeted. This knowledge helps bridge the gap between the accepted understanding of FIP as an immune-mediated inflammatory disease and the seemingly limited contribution of primary immune organs to inflammatory cytokine production. It also provides avenues for further study into the role of these organs in other feline systemic diseases.

## Figures and Tables

**Table 1:** Signalment and lesion distribution of cats in Group 1 (naturally and experimentally infected cats with feline infectious peritonitis (FIP) used for the liver study).

Cat	Group	Signalment	Lesion Distribution	Presence of Effusions
1	1.1a	6 m, MN, DSH	Peritoneum	Y
2	1.1a	6 m, FE, DSH	Peritoneum	Y
3	1.1a	1.5 y, FN, DLH	Kidney, lung	Y
4	1.1a	5 m, ME, DSH	Kidney, eye, brain	N
5	1.1a	Juvenile, FE, BSH	Kidneys, liver, spleen, lung, CNS	N
6	1.1a	Juvenile, MN, DSH	Liver, spleen, lungs, peritoneum, pleura	N
7	1.1a	8 m, MN, Burmese	Lung, kidney, brain, eye	N
8	1.1a	1 y, FN, DSH	Lung, brain	N
9	1.1a	2 y, MN, DSH	Lung, kidney, liver, peritoneum	N
10	1.1a	2 y, FE, DSH	NR	NR
11	1.1a	3 y, FN, DLH	NR	NR
12	1.1a	4 y, MN, DSH	NR	NR
13	1.1b	14–16 w, FE, DSH	Heart, lungs, spleen	N
14	1.1b	14–16 w, FE, DSH	Peritoneum, liver, kidney, omentum, spleen	Y
15	1.1b	14–16 w, FE, DSH	Peritoneum, liver, kidney, omentum, spleen	Y
16	1.1b	14–16 w, FE, DSH	Peritoneum, liver, omentum, spleen	Y

m: months; y: years; ME: male entire; MN: male neutered; FE: female entire; CNS: brain and spinal cord; N: no effusions; Y: effusions present; blank: data not available; NR: not recorded.

**Table 2:** Signalment and lesion distribution of cats in Group 2.

Cat	Group	Signalment	Lesion Distribution */Disease ^†^	Presence of Effusions
1	2.1	4 m, ME, Birman	Pleuritis, peritonitis, med ln	Y
2	2.1	7 m, ME, Devon Rex	Peritoneum, mes ln	Y
3	2.1	7 m, MN, BSH	Peritoneum, lung, liver, kidney, mes ln	Y
4	2.1	9 m, FN, DSH	Peritoneum, liver, mes ln, kidney	Y
5	2.1	1 y, MN, BSH	Pleura, liver, kidney, lung, mes ln	Y
6	2.1	11 m, ME, Birman	Pleura, peritoneum, liver, mes ln	Y
7	2.1	11 m, FN, Ragdoll	Peritoneum, intestinal wall, mes ln	Y
8	2.1	1 y, FN, DSH	Pleura, peritoneum, leptomeninx, lung, liver, kidneys	Y
9	2.1	1 y, MN, DLH	Peritoneum, liver, spleen, mes ln	Y
10	2.1	1 y, MN, Birman	Kidney, colon (BALT), liver, ln	Y
11	2.1	2 y, MN, DSH	Peritoneum, mes ln	Y
12	2.1	2 y, ME, British Blue	Widespread visceral lesions	Y
13	2.1	3 y, MN, Siamese	Pleuritis, peritoneum	Y
14	2.1	3 y, ME, Birman	Pleura	Y
15	2.1	4 m, ME, DSH	Liver, kidney, mes ln	N
16	2.1	8 m, MN, Ragdoll	Kidney, liver	N
17	2.1	9 m, ME, Birman	Brain	N
18	2.1	1 y, FN, DLH	NR	NR
19	2.2a	2 y, FN, DSH	Nasal polyp	
20	2.2a	2 y, FN, DSH	Oesophageal stricture	
21	2.2a	3 y, FN, DSH	Vertebral disc prolapse	
22	2.2a	3 y, MN, DLH	Lymphocytic cholangiohepatitis	
23	2.2b	9 y, FN, DSH	Unknown, no lesions	
24	2.2b	10 y, FN, DLH	Behavioural	
25	2.2b	10 y, FN, DSH	Behavioural	
26	2.2b	14 y, ME, DSH	Age-related	
27	2.2b	14 y, FN, DSH	Nasal osteosarcoma	
28	2.2b	19 y, FN, DSH	Gastrointestinal stromal tumour	
29	2.3	5 y, FN, Oriental Shorthair	Chlamydial infection	
30	2.3	5 y, MN, Oriental Shorthair	Chlamydial infection	
31	2.3	11 y, FN, DLH	Septic peritonitis	

* in the case of FIP; ^†^ in all non FIP cats; m: months; y: years; ME: male entire; MN: male neutered; FE: female entire; mes: mesenteric; med: mediastinal; ln: lymph node.

**Table 3:** Results of statistical comparisons (*p* values of a two-tailed Mann–Whitney) between cytokine mRNA transcription in the livers of naturally and experimentally infected cats with FIP and of experimentally FCoV-infected, healthy cats (FCoV-infected cats without FIP).

	*p* Values for Each Cytokine; * = Significant at 95% CI				
Group Comparison	IL-1β	IL-6	IL-10	IL-12p40	TNF-α
1.1a vs. 1.1b	0.103	0.770	0.170	0.133	0.078
1.1 vs. 1.2	0.002 *	0.001 *	0.034 *	0.001 *	0.017 *
1.1a vs. 1.2	0.000 *	0.003 *	0.031 *	0.002 *	0.004 *
1.1b vs.1. 2	0.721	0.012 *	0.382	0.061	0.878
1.1 eff vs. 1.1 no eff	0.628	0.035 *	1.000	0.836	0.234
1.1 eff vs. 1.2	0.274	0.020 *	0.312	0.009 *	0.494
1.1 no eff vs. 1.2	0.003 *	0.025 *	0.224	0.067	0.046 *

In all cases where the difference is significant, the first of the pair is higher. 1.1a: natural FIP cases; 1.1b: experimental FIP cases; 1.2: controls cats; 1.1 eff: FIP cats with effusions; 1.1 no eff: FIP cats without effusions.

**Table 4:** Results of statistical comparisons (*p* values of a two-tailed Mann–Whitney) between cytokine mRNA transcription in the hearts of naturally infected cats with FIP, cats with non-inflammatory conditions, and of cats with other systemic inflammatory diseases.

	*p* Values for Each Cytokine; * = Significant at 95% CI		
Group Comparison	IL-1β	IL-6	TNF-α
2.1 vs. 2.2	0.008 *	0.000 *	0.000 *
2.1 vs. 2.2a	0.165	0.122	0.000 *
2.1 vs. 2.2b	0.000 *	0.000 *	0.000 *
2.1 vs. 2.3	0.474	0.355	0.614
2.1 eff vs. 2.1 no eff	0.256	0.885	0.155
2.1 eff vs. 2.2	0.001 *	0.000 *	0.000 *
2.1 eff vs. 2.2a	0.410	0.093	0.000 *
2.1 eff vs. 2.2b	0.000 *	0.000 *	0.000 *
2.1 eff vs. 2.3	0.886	0.255	0.437
2.1 no eff vs. 2.2	0.331	0.018 *	0.001 *
2.1 no eff vs. 2.2a	0.159	0.332	0.025 *
2.1 no eff vs. 2.2b	0.013 *	0.002 *	0.000 *
2.1 no eff vs. 2.3	0.217	0.529	0.894

In all cases where the difference is significant, the first of the pair is higher. 2.1: cats with FIP; 2.2: control cats; 2.2a: control cats up to 3 years old; 2.2b: control cats greater than 9 years old; 2.3: cats with systemic inflammatory disease other than FIP; 2.1 eff: FIP cats with effusions; 2.1 no eff: FIP cats without effusions.

## Supplementary Materials

The following are available online at https://www.mdpi.com/1999-4915/11/12/1144/s1, Table S1: Amino acid sequences used for anti-feline cytokine antibody production, Table S2: Distribution statistics for all liver study groups, Table S3: Distribution statistics for all heart study groups.
