# Supplementary material for: Feline Infectious Peritonitis as a Systemic Inflammatory Disease: Contribution of Liver and Heart to the Pathogenesis
Source: Viruses. 2019 Dec 10;11(12):1144. doi: 10.3390/v11121144 (PMC6949997; doi:10.3390/v11121144)
Supplement: Supplementary file 1 [file viruses-11-01144-s001.zip › viruses-644192-suppl/Table S1.pdf]

Table S1: Amino acid sequences used for anti-feline cytokine antibody production.

Cytokine	NCBI Acc.-No.	Position	Amino acid sequence
IL-1β	AAA30814	251-264	Cys-NTKGGQDITDFIME
IL-6	P41683	191-204	Cys-LRRLEDFLQFSLRA
IL-10	AAC64708	144-157	Cys-STFSKLQEKGVYKA
IL-12p40	AAB93835	185-198	Cys-RDYKKYTVECQEGS
TNF-α	AAA30818	202-215	Cys-LEKGDRLSAEINLP
