# Supplementary material for: Feline Infectious Peritonitis as a Systemic Inflammatory Disease: Contribution of Liver and Heart to the Pathogenesis
Source: Viruses. 2019 Dec 10;11(12):1144. doi: 10.3390/v11121144 (PMC6949997; doi:10.3390/v11121144)
Supplement: Supplementary file 1 [file viruses-11-01144-s001.zip › viruses-644192-suppl/Table S2.pdf]

Table S2: Distribution statistics for all liver study groups.

Cytokine	Group	No.+ve/ group total	Distribution statistics				
			Mean ($2^{-\Delta CT}$)	SE of mean	Median ($2^{-\Delta CT}$)	SD	Variance
IL-6	1.1	14/16	0.052	0.046	0.005	0.186	0.034
	1.1a	10/12	0.067	0.062	0.003	0.214	0.046
	1.1b	4/4	0.008	0.004	0.006	0.009	0.000
	1.1eff	5/6	0.008	0.003	0.007	0.008	0.000
	1.1no eff	6/7	0.001	0.000	0.000	0.002	0.000
	1.2	12/14	0.000	0.000	0.000	0.000	0.000
IL-10	1.1	13/16	0.011	0.004	0.004	0.015	0.000
	1.1a	9/12	0.014	0.005	0.008	0.016	0.000
	1.1b	4/4	0.001	0.000	0.001	0.001	0.000
	1.1eff	5/6	0.010	0.007	0.001	0.018	0.000
	1.1no eff	5/7	0.008	0.004	0.002	0.013	0.000
	1.2	14/14	0.000	0.000	0.000	0.000	0.000
IL-12p40	1.1	15/16	0.007	0.003	0.004	0.011	0.000
	1.1a	11/12	0.010	0.003	0.009	0.009	0.000
	1.1b	4/4	0.000	0.000	0.000	0.000	0.000
	1.1eff	6/6	0.004	0.004	0.000	0.009	0.000
	1.1no eff	6/7	0.010	0.005	0.006	0.015	0.000
	1.2	14/14	0.000	0.000	0.000	0.000	0.000
TNF-α	1.1	14/16	0.008	0.002	0.007	0.009	0.000
	1.1a	10/12	0.010	0.003	0.009	0.009	0.000
	1.1b	4/4	0.000	0.000	0.000	0.000	0.000
	1.1eff	5/6	0.002	0.002	0.001	0.004	0.000
	1.1no eff	6/7	0.007	0.002	0.007	0.006	0.000
	1.2	13/14	0.000	0.000	0.000	0.000	0.000

1.1a: natural FIP cases; 1.1b: experimental FIP cases; 1.2: controls cats; 1.1eff: FIP cats with effusions; 1.1no eff: FIP cats without effusions; +ve: positive by PCR for the given cytokine; SE: standard error; SD: standard deviation.
