# Supplementary material for: Feline Infectious Peritonitis as a Systemic Inflammatory Disease: Contribution of Liver and Heart to the Pathogenesis
Source: Viruses. 2019 Dec 10;11(12):1144. doi: 10.3390/v11121144 (PMC6949997; doi:10.3390/v11121144)
Supplement: Supplementary file 1 [file viruses-11-01144-s001.zip › viruses-644192-suppl/Table S3.pdf]

Table S3: Distribution statistics for all heart study groups.

Cytokine	Group	Distribution statistics				
		Mean ($2^{-\Delta CT}$)	SE of mean	Median ($2^{-\Delta CT}$)	SD	Variance
IL-1β	2.1	9.29E-05	8.60E-06	8.20E-05	7.53E-05	5.67E-05
	2.1eff	1.06E-04	9.60E-06	1.05E-04	7.06E-05	4.98E-05
	2.1no eff	7.57E-05	1.99E-05	6.50E-05	8.20E-05	6.72E-05
	2.2	5.32E-05	1.18E-05	5.40E-05	8.07E-05	6.51E-05
	2.2a	1.20E-04	1.49E-05	1.20E-04	6.32E-05	4.00E-05
	2.2b	1.18E-05	1.12E-05	2.00E-05	6.03E-05	3.63E-05
	2.3	1.03E-04	1.30E-05	1.06E-04	4.30E-05	1.85E-05
IL-6	2.1	1.72E-04	9.60E-06	1.71E-04	8.40E-05	7.05E-05
	2.1eff	1.80E-04	1.09E-05	1.81E-04	8.23E-05	6.78E-05
	2.1no eff	1.62E-04	2.26E-05	1.54E-04	9.03E-05	8.16E-05
	2.2	9.67E-05	1.38E-05	7.05E-05	9.39E-05	8.82E-05
	2.2a	1.39E-04	2.56E-05	1.12E-04	1.09E-04	1.18E-04
	2.2b	6.93E-05	1.37E-05	6.05E-05	7.27E-05	5.29E-05
	2.3	1.49E-04	2.32E-05	1.41E-04	7.32E-05	5.36E-05
TNF-α	2.1	9.95E-05	7.60E-06	9.10E-05	6.79E-05	4.61E-05
	2.1eff	1.09E-04	1.03E-05	1.12E-04	7.52E-05	5.65E-05
	2.1no eff	8.74E-05	1.07E-05	7.85E-05	5.00E-05	2.50E-05
	2.2	1.07E-05	1.11E-05	1.10E-05	7.42E-05	5.51E-05
	2.2a	1.93E-05	1.86E-05	3.70E-05	7.44E-05	5.54E-05
	2.2b	5.90E-06	1.39E-05	4.00E-06	7.50E-05	5.62E-05
	2.3	9.82E-05	2.57E-05	6.95E-05	8.89E-05	7.91E-05

2.1: cats with FIP; 2.2: control cats; 2.2a: control cats up to 3 years old; 2.2b: control cats greater than 9 years old; 2.3: cats with systemic inflammatory disease other than FIP; 2.1eff: FIP cats with effusions; 2.1no eff: FIP cats without effusions.
